# Efficacy and safety of second-line fotemustine in elderly patients with recurrent glioblastoma

**DOI:** 10.1007/s11060-013-1125-3

**Published:** 2013-04-06

**Authors:** Matteo Santoni, Silvia Scoccianti, Ivan Lolli, Maria Grazia Fabrini, Giovanni Silvano, Beatrice Detti, Franco Perrone, Giuseppina Savio, Roberto Iacovelli, Luciano Burattini, Rossana Berardi, Stefano Cascinu

**Affiliations:** 1 Clinica di Oncologia Medica, AOU “Ospedali Riuniti”, Università Politecnica delle Marche, via Tronto 10/A, 60100 Ancona, Italy; 2 Azienda Ospedaliera Universitaria Careggi, Florence, Italy; 3 Oncologia Medica, IRCCS “Saverio de Bellis”, via Turi 27 Castellana Grotte, 70013 Bari, Italy; 4 Azienda Ospedaliero-Universitaria Pisana, Pisa, Italy; 5 Radioterapia Oncologica SG Moscati Hospital, Taranto, Italy; 6 U.O. Oncologia Medica ARNAS Civico, Palermo, Italy; 7 Dipartimento di Scienze Radiologiche, Oncologiche e Anatomo-Patologiche, “Sapienza” Università di Roma, Rome, Italy

**Keywords:** Elderly patients, Fotemustine, Glioblastoma, Safety, Temozolomide

## Abstract

Fotemustine (FTM) is a common treatment option for glioblastoma patients refractory to temozolomide (TMZ). Although elderly patients represent a large component of glioblastoma population, the feasibility and the efficacy of second-line FTM are not available in those patients.We retrospectively analyzed the records of glioblastoma patients older than 65 years, receiving FTM at a dose of 70–100 mg/m^2^ of FTM every week for 3 consecutive weeks (induction phase) and then every 3 weeks (70–100 mg/m^2^), as second-line treatment.Between January 2004 and December 2011, 65 glioblastoma patients (median age, 70 years; range, 65–79 years) were eligible for this analysis. Sixty-five patients received a total of 364 FTM cycles, with a median of 4 cycles for each patient. After induction, we observed 1 complete response (1.5 %), 12 partial responses (18.5 %), 18 stable diseases (27.7 %), and 34 patients’ progressions (47.7 %). Disease control rate was 43.1 %. Median survival from the beginning of FTM therapy was 7.1 months, while the median progression-free survival was 4.2 months, and the 6-months progression free survival rate was 35.4 %. The most relevant grade 3–4 toxicity events were thrombocytopenia (15.3 %) and neutropenia (9.2 %). In the univariate and multivariate analysis, time from radiotherapy to FTM, number of TMZ and FTM cycles and disease control resulted independent prognostic factors.This study showed that FTM is a valuable therapeutic option for elderly glioblastoma patients, with a safe toxicity profile.

## Introduction

Glioblastoma multiforme (GBM) is the most common type of adult primary central nervous system tumour, accounting for 50 % of gliomas [1]. While elderly patients constitute a large component of the GBM population, the optimal management of elderly patients still remains controversial. In fact, no evidence-based standard of care exists for this unique subpopulation that is often excluded from clinical trials. Survival of elderly GBM patients is poor, probably due to the reduced use of standard management approaches, increased toxicity of available therapies, and increased presence of comorbidities in this older patient population.

The advantage of debulking surgery remains unknown in this fragile cohort of patients. Elderly patients commonly receive temozolomide (TMZ) or radiotherapy (RT), even if most of them are candidate for palliative approaches following surgical diagnosis [2–5]. Concurrent TMZ chemotherapy during RT improves the survival of younger patients with GBM [6], but the benefit in elderly patients is unclear, although a growing body of evidence suggests that fit elderly patients benefit from the addition of TMZ to standard surgery and radiation [7–12].

Even more debatable is the role of a second-line chemotherapy. Fotemustine (FTM), a third generation chloroethylnitrosourea, has been investigated in malignant glioma patients recurring after TMZ standard treatment [13–17]. However, in these trials data on the efficacy and feasibility in elderly patients are not available.

We retrospectively evaluated the efficacy and toxicity of second-line FTM in elderly patients with recurrent GBM treated with prior radiotherapy and TMZ, as well as specific independent predictors of survival.

## Methods

Seven Italian centers were involved in this retrospective analysis. Patients older than 65 years with recurrent or progressive, histologically-confirmed GBM, previously treated with surgery and RT plus concomitant and adjuvant TMZ, were included in the analysis. They received 1 h intravenous infusion of FTM according to the following schedule: induction phase dose of 70–100 mg/m^2^ on days 1, 8, 15, followed by a 4/5-week rest period, and a maintenance phase dose of 70–100 mg/m^2^ every 21 days. Toxicity was graded according to the National Cancer Institute (NCI) common toxicity criteria (CTC, version 3.0).

Baseline MRI or CT neuro-imaging was performed before administration of FTM, and subsequent evaluations were carried out after completion of the induction phase, every two cycles during the maintenance phase, according to Macdonald’s criteria [18]. Steroid dosing was considered during radiographic disease assessment. In the presence of complete or partial responses (CR and PR) or stable disease (SD), the time to progression (TTP) was evaluated until progressive disease (PD), even if the treatment was discontinued. Disease control (DC) was defined as CR + PR + SD. Overall survival (OS) was measured from the start of FTM to death for any reason, or last follow-up assessment. Neurological status was assessed by considering signs and symptoms possibly correlated with progression, as compared with the previous examination.

The endpoints of this analysis were: toxicity profile, DC, progression-free survival rate at 6 months (PFS-6) and at 1 year (PFS-1y), OS from the diagnosis and from the beginning of FTMS, overall survival rate at 1 year (OS-1y) and TTP.

Cox proportional hazards models were applied to explore patients’ characteristics predictors of survival in univariate- and multivariable-adjusted analysis using a stepwise selection approach with type I error of 0.05 for model entry and 0.10 for elimination. Additional elimination was applied to identify significant variables at the level of *P* < 0.05. We used PASW (Predictive Analytics SoftWare) (v 18; IBM SPSS).

## Results

Between January 2004 and December 2011, among the 206 GBM patients receiving FTM as second-line treatment after surgery and concomitant radio-chemotherapy plus adjuvant TMZ failure, 65 patients (35 males and 30 females) older than 65 years, were included in this analysis. The majority of them had Karnofsky Performance Status (KPS) >70. The demographic and clinical characteristics of patients are outlined in Table 1.

**Table 1:** Patients’ characteristics

Patients	65
Gender	
Female	30
Male	35
Age (years)	
Median	70
65–70	30
70–75	31
>75	4
Karnofsky performance status	
90–100	24
70–80	38
<70	3
Extent of resection	
Gross total resection	53
Partial resection or biopsy	12
Cycles of adjuvant temozolomide	
Median	6
Range	2–24
Cycles of fotemustine	
Median	4
Range	2–20

All patients received at least one dose of FTM. Forty-one patients (63.1 %) started maintenance chemotherapy, with a median of 4 cycles received after the induction phase (range 1–17). Eight patients (12.3 %) did not start maintenance therapy because of a disease progression.

One complete response (1.5 %), 12 partial responses (18.5, 95 % CI: 9.1–27.9 %), 18 stable disease (27.7, 95 % CI: 16.8–38.6 %), and 34 patients’ progressions (52.3, 95 % CI: 35.6–59.8 %) were obtained after FTM induction. Median duration of disease stabilization was 7.3 months (95 % CI: 4.5–10.1). DC was registered in 43.1 % of patients.

The median OS from the start of FTM treatment was 7.1 months (95 % CI, 1.6–35.9) and the 1y-OS rate was 20.0 % (95 % CI 10.3–29.7 %).

**Fig. 1:**
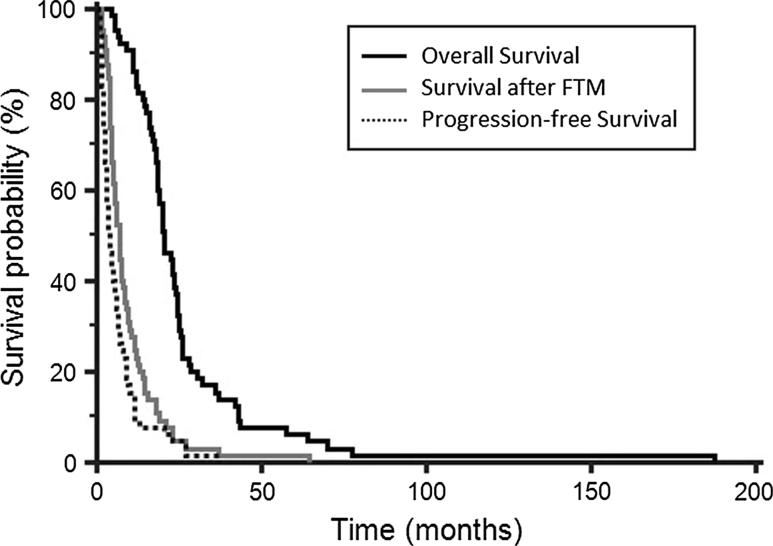
Overall survival (OS), survival after fotemustine and progression-free survival (PFS) in elderly glioblastoma patients treated with fotemustine as second-line therapy

The median TTP for FTM was 4.2 months (95 % CI 1.2–24.1 months) (Fig. 1), with a PFS-6 rate of 35.4 % (95 % CI 23.8–47.0 %) and PFS-1y of 13.8 % (95 % CI 5.4–22.8 %). Results are summarized in Table 2.

**Table 2:** Results obtained by using fotemustine as second-line therapy in elderly patients with recurrent glioblastoma

Objective responses	
Complete responses (CR)	1 (1.5 %)
Partial responses (PR)	12 (18.5 %)
Stable diseases (SD)	18 (27.7 %)
Progressive diseases (PD)	34 (52.3 %)
Disease control (DC)	43.1 %
Median overall survival (months)	20.6
Median overall survival from II line fotemustine (months)	7.1
Progression free survival (months)	4.2
Six months-progression free survival (%)	35.4 %
One year-progression free survival (%)	13.8 %

Fourteen patients (21.5 %) with progressive disease after FTM and acceptable general conditions underwent a third line chemotherapy.

All 65 patients exposed to FTM were evaluated for safety. FTM administration was well tolerated and the most relevant grade 3–4 toxicity events were thrombocytopenia (15.3 %) and neutropenia (9.2 %) (Table 3). Only one patient, with prolonged grade 2 thrombocytopenia, discontinued therapy due to toxicity during the induction phase. None of the patients manifested constitutional or neurological symptoms during FTM treatment. No significant differences in terms of incidence of hematologic toxicities were found in patients stratified by age (>65/>70/>75).

**Table 3:** Incidence of drug-related adverse events during II line treatment with fotemustine in elderly patients with recurrent glioblastoma

Thrombocytopenia	*N* (%)
Grade 1–2	9 (13.8)
Grade 3–4	10 (15.3)
Leukopenia	
Grade 1–2	11 (16.9)
Grade 3–4	6 (9.2)
Neutropenia	
Grade 1–2	7 (10.8)
Grade 3–4	6 (9.2)
Lymphopenia	
Grade 1–2	6 (10.0)
Grade 3–4	5 (7.7)
Anemia	
Grade 1–2	4 (6.2)
Grade 3–4	2 (3.1)
Transaminase elevation	
Grade 1–2	5 (7.7)
Grade 3–4	3 (4.6)

Furthermore, we performed an univariate and multivariate analysis to evaluate the impact of gender, time from RT to FTM, number of TMZ and FTM cycles and DC on OS and PFS of elderly patients. In univariate analysis, time from RT to FTM (*P* < 0.001), number of TMZ (*P* = 0.001) and FTM cycles (*P* < 0.001) and DC (*P* < 0.001) had a significant impact on OS, whereas only time from RT to FTM and number of TMZ cycles significantly affected PFS. Multivariate analysis showed time from RT to FTM (*P* < 0.001), number of TMZ (*P* < 0.001) and FTM cycles (*P* < 0.001) and DC (*P* = 0.021) for OS and time from RT to FTM (*P* = 0.03) and number of TMZ cycles (*P* < 0.001) for PFS as independent prognostic factors (Tables 4, 5).

**Table 4:** Univariate and multivariate analysis of OS in elderly patients treated with fotemustine for recurrent glioblastoma

OS	Univariate Cox regression		Multivariable Cox regression	
	HR (95 %CI)	*p* value	HR (95 %CI)	*p* value
Sex (m/f)	1.07 (0.63–1.80)	0.809		
N° cycles of TMZ (cont)	0.89 (0.84–0.95)	0.001	0.77 (0.68–0.88)	<0.001
Time from RT to FTM	0.89 (0.84–0.93)	<0.001	0.90 (0.85–0.95)	<0.001
N° cycles of FTMS (cont)	0.35 (0.20–0.61)	<0.001	0.84 (0.78–0.91)	<0.001
Disease control (*n*/*y*)	2.72 (1.56–4.75)	<0.001	2.03 (1.11–3.69)	0.021

*OS* overall survival, *HR* hazard ratio, *CI* confidence interval, *TMZ* temozolomide, *FTM* fotemustine, *N°* number, *cont* continuous variable, *RT* radiotherapy

**Table 5:** Univariate and multivariate analysis of PFS in elderly patients treated with fotemustine for recurrent glioblastoma

PFS	Univariate Cox regression		Multivariable Cox regression	
	HR (95 %CI)	*p* Value	HR (95 %CI)	*p* value
Sex (m/f)	1.29 (0.79–2.11)	0.311		
Time from RT to FTM	0.95 (0.92–0.99)	0.008	0.95 (0.91–0.99)	0.03
N° cycles of TMZ (cont)	0.87 (0.81–0.93)	<0.001	0.87 (0.81–0.93)	<0.001

*OS* overall survival, *HR* hazard ratio, *CI* confidence interval, *TMZ* temozolomide, *FTM* fotemustine, *N°* number, *cont* continuous variable, *RT* radiotherapy

## Discussion

Recurrent GBM is resistant to most therapeutic approaches and elderly patients (>65 years old) have a significantly worse life expectancy compared with younger patients. In addition, no evidence-based standard of care exists for this unique subpopulation (Table 6).

**Table 6:** Results with the use of second-line fotemustine in glioblastoma patients

Authors	Years	Patients enrolled	Median PFS	PFS-6 (%)
Malhaire et al. [13]	1999	22	6.5	NR
Scoccianti et al. [15]	2008	27	5.7	48.2
Brandes et al. [7]	2009	43	NR	20.9
Fabrini et al. [16]	2009	50	6.1	51.5
Paccapelo et al. [17]	2012	163	NR	25.0–43.8

*OS* overall survival, *PFS* progression-free survival, *PFS-6* progression-free survival at 6 months, *NR* not reported

Elderly patients with GBM represent a major focus in neuro-oncology. Recently, Malmström et al. have led a phase III study of GBM patients older then 60y, randomized to receive TMZ vs. standard 6-week radiotherapy versus hypofractionated radiotherapy. The results showed that standard radiotherapy was associated with poor outcomes, suggesting to consider both TMZ and hypofractionated radiotherapy as standard treatment options in elderly patients with GBM [19]. Additionally, Wick et al. has recently reported that TMZ alone is non-inferior to radiotherapy alone in the treatment of elderly patients with malignant astrocytoma [20].

FTM has been demonstrated to represent a valid option for patients with recurrent or progressive GBM. Several trials [13–17] have investigated the use of FTM in recurrent GBM patients, with the PFS-6 ranging from the 20 % reported by GICNO [14] to the 48 and 51.5 % reported, respectively by Scoccianti et al. [15] and Fabrini et al. [16] (Table 6). In these studies, myelosuppression was the most common adverse event that occurred, mainly during the induction phase of treatment and more frequently in TMZ pretreated patients.

The comparison between the results reported by Paccapelo [17] and Perry [21] could suggest a potentially different response pattern between recurrent GBM patients treated with FTM and those with rechallenge TMZ. Indeed, TMZ seemed to be active in early and late progression patients, while FTM was always active in recurrent patients. The major difference was registered in GBM patients who failed after more than 6 months of TMZ [17].

As regard to the use of bevacizumab alone or in combination with irinotecan in GBM patients in first or second relapse, Friedman et al showed an estimated PFS-6 of 42.6% and 50.3%, respectively. However, in this study median age was < 60 in the two groups, and data on the elderly patients were not reported in the results [22].

Our retrospective study represents the first report on efficacy and safety of FTM in elderly patients with recurrent GBM. In our analysis, FTM was a valuable therapeutic option for elderly patients with recurrent GBM, obtaining a median OS of 7.1 months, a PFS-6 of 35.4 % and a median PFS of 4.2 months. In addition, FTM administration was feasible with an acceptable toxicity, even in this group of patients.

When stratified by age category (>65/>70/>75), no significant differences were registered in the incidence of hematologic adverse events, although this analysis is limited by the small number of patients >75y in our study.

Furthermore, in our study population, time from RT to FTM, number of TMZ and FTM cycles and DC resulted independent treatment-related prognostic factors. It is also of interest the high percentage (21.5 %) of patients still fit for third line chemotherapy after FTM failure.

However, elderly GBM patients fit for second-line therapies are good prognosis patients, and our data in terms of tolerance and efficacy of FTM may not be extended to the overall elderly GBM population.

In conclusion, FTM may be considered as a treatment option, even for elderly GBM patients, especially for those receiving a significant benefit from prior TMZ therapy and with good KPS. Based on these data these patients should definitely be included in adult patients clinical trials.
